# The burden of malaria in Sudan: incidence, mortality and disability – adjusted life – years

**DOI:** 10.1186/1475-2875-6-97

**Published:** 2007-07-28

**Authors:** Safa I Abdalla, Elfatih M Malik, Kamil M Ali

**Affiliations:** 1 Department of Community Medicine, Faculty of Medicine, University of Khartoum, Khartoum, Sudan; 2 National Malaria Control Programme, Federal Ministry of Health, Khartoum, Sudan

## Abstract

**Background:**

Estimating the burden of malaria in Sudan is important for evidence-based planning of malaria control. Estimates of malaria burden in terms of DALYs (Disability Adjusted Life Years) were not developed locally. This study synthesized information from different sources to calculate malaria incidence, mortality and DALYs lost in Sudan in 2002.

**Methods:**

A search for local studies and reports providing epidemiological data on malaria in Sudan was conducted. Preliminary estimates of incidence rate, case fatality rate and mortality rate were developed from the data found. The preliminary estimates were processed in the disease modelling computer software, DisMod II, to produce internally consistent mortality and incidence rates, which were used to calculate DALYs lost due to malaria.

**Results:**

Malaria incidence in Sudan was estimated to be about 9 million episodes in 2002 and the number of deaths due to malaria was about 44,000. 2,877,000 DALYs were lost in Sudan in 2002 due to malaria mortality, episodes, anaemia and neurological sequelae. Children under five years of age had the highest burden. Males had the highest incidence and mortality, but females lost more DALYs.

**Conclusion:**

Formal health system data underestimated malaria burden. The burden estimates can be useful in informing decision making, although uncertainty around them needs to be quantified. Epidemiological research is needed to fill data gaps and update the estimates.

## Background

Estimating the burden of malaria is highly needed for evidence based planning of malaria control. In Sudan, malaria has been the subject of a large amount of epidemiological, entomological and biomedical research. Malaria surveillance, as part of the general reporting of health events from health facilities or specific surveillance for epidemic preparedness, provided a wide range of information. This resulted in multiple and diverse sources of information about malaria burden in Sudan, each source serving the purpose for which it was established. The problems with these sources are non-representativeness, variability of the sensitivity and specificity of the diagnostic criteria used and variability of the indices measured. Some may suffer from underreporting. These sources, therefore, could not directly provide a single valid national indicator of malaria burden. Official figures of incidence and mortality reflected cases reported only from the formal health system. Malaria health burden results from both disability and mortality, so that multiple statistics are needed to describe it. DALYs (Disability Adjusted Life Years) are composite indicators that summarize disability and mortality information in a single number [1]. The DALYs approach would be suitable to express the burden of malaria resulting from fatal episodes as well as non fatal episodes and complications. It would also act as a common index to compare the burden of malaria with that of other diseases. Previous attempts to calculate the burden of malaria in Sudan were made as part of the GBD (Global Burden of Disease) study using different indirect methods [2,3].

No local efforts have previously been made to measure the burden of malaria in terms of DALYs in Sudan, using locally-derived epidemiological data. As part of a project to measure the burden of diseases in Sudan, this study synthesized information about the epidemiology of malaria from different sources to calculate national incidence rate, mortality rate and DALYs lost due to malaria.

## Methods

The study measured the burden of malaria in 2002 for all Sudan. The burden was attributed to mortality and disability from malaria episodes, anaemia and neurological sequelae, as defined in the GBD 2002 [2]. Multiple levels of malaria endemicity are found in Sudan [4], and calculations were based on classification of the 26 states of Sudan into hypoendemic, mesoendemic and hyperendemic areas.

### Data collection

Relevant published and unpublished studies were found by electronic search in local databases and Pubmed, using the keywords: malaria, Sudan, Africa, case fatality, age distribution, sex distribution, anaemia, cerebral malaria, and neurological sequelae. Postgraduate thesis archives were hand-searched for thesis not indexed in local databases. Research institutes, governmental and non-governmental organizations were contacted and relevant reports obtained. Reference lists were examined for more research papers. Studies and reports were relevant if they provided at least one of the following: incidence, prevalence, mortality rate, case fatality ratio, age or sex distribution of cases or deaths, remission proportion, average duration and disability proportions. There was no restriction regarding the time of study. Studies identified were reviewed systematically and those selected for use in the calculations were the most representative and with the best methodological quality where applicable.

### Demographic variables

The total population by age and sex for Sudan in 2002 was interpolated from the 1998 and 2003 population projections of the Sudan Central Bureau of Statistics [5]. Age- and sex-specific all cause mortality rates were obtained from life tables developed by the World Health Organization (WHO) for Sudan in 2002 using indirect demographic techniques [6]. Total population estimates were used to calculate rates and together with all cause mortality rates served as input for computer based disease modelling.

### Preliminary incidence of malaria

**Figure 1:**
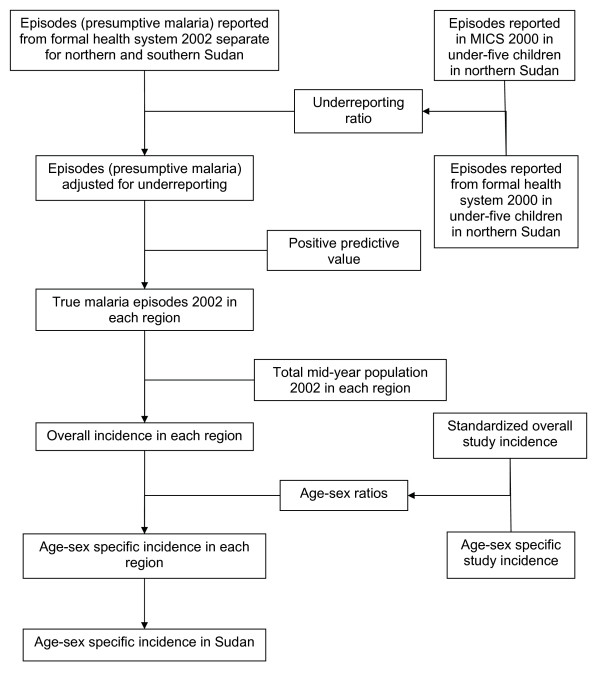
**Malaria incidence calculation model**. Malaria underreporting ratio was calculated using reported episodes in 2000 and results of MICS (Multiple Indicators Cluster Survey) 2000. The number of reported episodes of presumptive malaria in 2002 in northern and southern Sudan was corrected for underreporting, and adjusted downwards to calculate true malaria incidence in each region. Age-sex ratios were used to disaggregate the overall incidence in each region and the incidence in each age-sex group was pooled to estimate the age-sex specific incidence in all Sudan.

Figure 1 shows the model used to calculate preliminary malaria incidence rates by age and sex. Completeness of reporting of malaria episodes to the formal health system in 2000 [7] was calculated using Multiple Indicators Cluster Survey (MICS) results for children < 5 years as gold standard [8]. The latter referred to fever episodes for which an antimalarial was prescribed, in the two weeks prior to the survey, while the reported episodes referred to clinically or laboratory confirmed episodes. The calculations were therefore assumed to refer to presumptive malaria. The annual reported episodes number was divided by 26 to derive the average number of episodes in two weeks. The MICS number of episodes was divided by that average to calculate an underreporting ratio which was applied to the annual reported number of episodes in northern Sudan (16 states) and southern Sudan (10 states) in 2002 in all age groups [9,10]. Because of the variable sensitivity and specificity of laboratories in malaria diagnosis [11], the resulting incidence was adjusted downwards using the positive predictive value of fever and other malaria symptoms for a positive blood film, to obtain the true malaria incidence. Several published studies [12-14] and unpublished data (Institute of Endemic Diseases, Sudan) provided predictive values for different areas and seasons. The median of these values (0.3) was used for northern Sudan, while a higher value (0.5) was applied for southern Sudan, the area being hyperendemic for malaria [4]. Malaria surveillance in a village in northern Sudan reported age-sex specific incidence rates [15]. In southern Sudan, the age distribution of fever in the absence of neck stiffness (International Rescue Committee, Sudan, unpublished data) was used as a proxy for malaria age distribution. The age-sex specific rates were used to standardize the studies incidence rates using Sudan population in 2002 and the results used to disaggregate the overall incidence calculated for the two regions by age and sex. The rates for the two regions were combined to produce the national age-sex specific malaria incidence rates.

### Preliminary case fatality and mortality rates

**Figure 2:**
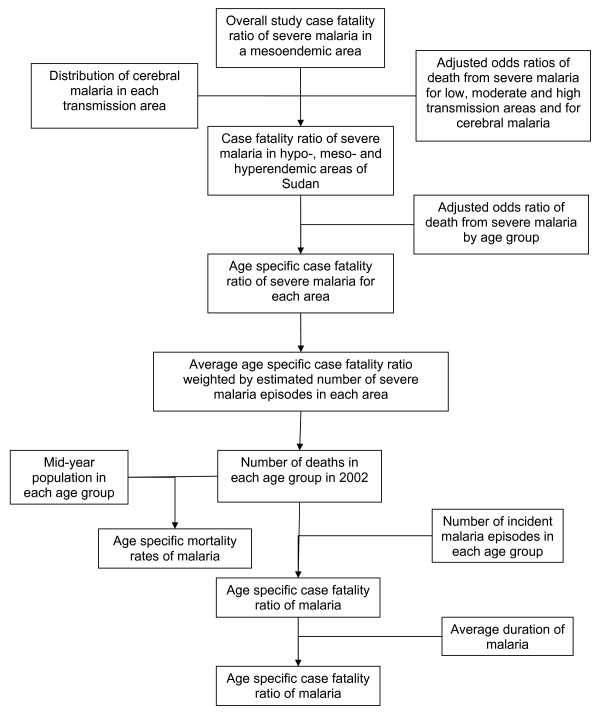
**Malaria case fatality and mortality calculation model**. Overall case fatality ratio of severe malaria reported in a mesoendemic area was used to extrapolate the case fatality ratio in hypoendemic and hyperendemic areas, after adjusting for differences in cerebral malaria proportions between the different endemicity areas. The overall ratios were disaggregated by age and pooled for all areas in each age group. The age specific number of deaths was used to calculate age specific mortality rates and was combined with the number of malaria episodes to calculate age specific case fatality ratios and rates of malaria.

Figure 2 shows the model used to calculate age specific case fatality and mortality rates. Using adjusted odds ratios of mortality of severe malaria in low, medium and high transmission areas [16], the case fatality ratios of severe malaria in hypoendemic and hyperendemic areas were extrapolated from that in a mesoendemic area [17], after adjusting for differences in cerebral malaria risk between the different areas. Adjusted odds ratios of mortality of severe malaria by age group were used to disaggregate the case fatality ratios by age. In each age group the ratios for all endemicity levels in Sudan were pooled using the estimated number of severe malaria episodes. The latter were the result of combining the age distribution of severe malaria [17], the proportion of total population in each area and the total number of malaria episodes according to the preliminary incidence rate. The number of deaths in each age group was computed and together with the mid-year population and average duration [17], was used to estimate mortality and case fatality rates of malaria.

### Development of internally consistent estimates

Age-sex specific preliminary incidence, mortality and case fatality rates were processed in DisMod II. This is a disease modelling software that uses input estimates to calculate an internally consistent set of epidemiological estimates [18]. The output incidence rates, mortality rates and average duration were used in estimating the epidemiology of neurological sequelae and anaemia, and in calculating DALYs.

### Neurological sequelae

Adjusted odds ratios of cerebral malaria [16] were used to extrapolate proportions of cerebral malaria in hypoendemic and hyperendemic areas from the proportion reported in a mesoendemic area [17]. Adjusted odds ratios of cerebral malaria by age were used to calculate age-specific proportions in each area. The overall proportions in each age group for Sudan were calculated in the same way as the case fatality ratios, and they were used to estimate the incidence of cerebral malaria. The incidence of neurological sequelae was derived by applying the proportion of cerebral malaria episodes that cause neurological sequelae. This proportion together with the remission rate of neurological sequelae was obtained from an African study [19]. Assuming zero case fatality, the average duration was then calculated in DisMod II using the incidence, case fatality and remission rates.

### Anaemia

The age-specific proportions of severe anaemia amongst severe malaria cases were calculated using adjusted odds ratios of severe anaemia in the same way as neurological sequelae. Studies reported the ratio of severe anaemia to mild and moderate anaemia [20-22], and the median of these ratios was applied to the proportion of severe anaemia to compute the proportion of episodes developing all degrees of anaemia. Incidence of anaemia was calculated from DisMod II incidence output and the proportion of episodes developing all degrees of anaemia. The case fatality ratio of severe anaemia in children [21] was used to derive the number of deaths due to severe anaemia. This, together with the number of anaemia episodes in 2002, was used to calculate the case fatality rate. Anaemia remission rate was computed assuming 100% remission of mild and moderate anaemia and 30% remission of severe anaemia in 4 weeks [23]. Age-sex specific incidence, remission and case fatality rates of malarial anaemia were processed in DisMod II to calculate the average duration.

### YLLs (Years of Life Lost), YLDs (Years Lived with Disability) and DALYs

According to GBD methods [18], DisMod II output incidence, mortality and average duration were used to calculate YLLS, YLDs and DALYs, using the following formulas:

• YLL = number of deaths X standard life expectancy at the average age of death

• YLD = number of incident cases/episodes X average duration X disability weights

• DALY = YLL + YLD

Standard life expectancies of the GBD 1990 [1] and disability weights of the GBD 2002 [2] were used.

## Results and discussion

### Incidence

Table 1 shows the age- and sex-specific incidence of malaria in Sudan in 2002. The overall incidence of malaria was 282 per 1,000 population and was higher in males than in females. The total number of episodes in 2002 from this study was about nine million, exceeding the number (3,054,400 episodes) reported from the formal health system [9]. Under-reporting was taken into account in the calculations in a way similar to that used to quantify malaria burden in two districts in southern Ghana [24]. However, some error would result from comparing the formal health system figures, where the biweekly incidence referred to an average for the whole year, with the results of a survey in a specific season of the year, considering the seasonality of malaria in some parts of Sudan. The underreporting ratio of presumptive malaria calculated was less than that found in southern Ghana (one case in the formal health system to four to five cases never registered), but was in agreement with the finding that less than 20% of cases in sub-Saharan Africa reach the formal health system [25]. Using the true malaria incidence, reporting completeness would be about 30%. However, Korenromp calculated malaria incidence globally and at regional and country level in 2004 [3], estimating a case reporting completeness of 85% for Sudan.

**Table 1:** Age and sex specific incidence of malaria per 1000 population in Sudan in 2002

Age group	Males	Females	Total
0–4	310	256	283
5–14	361	320	381
15–29	298	294	296
30–44	222	214	218
45–59	249	185	217
60–69	203	183	193
70–79	203	183	193
80+	203	183	193
Overall	297	267	282

Children 5 – 14 years had the highest incidence of malaria. The incidence declined afterwards to rise in males 45 – 59 years old. In all age groups, the incidence was higher in males than in females.

In both males and females the highest incidence was in children 5 – 14 years of age. It declined afterwards and increased again in males in the age group 45 – 59. The incidence was higher in males than in females in all age groups. These age and sex patterns reflect the original patterns in the studies used in estimation of the preliminary incidence rates.

### Mortality

The mortality rate of malaria was the same in males and females (Table 2). This was due to the assumption of equal case fatality and mortality rates in the two sexes. Mortality was highest in children less than five years old and declined in older age groups, reflecting the mortality age distribution constructed in the preliminary estimates.

**Table 2:** Age and sex specific mortality of malaria per 100,000 population in Sudan in 2002

Age group	Males	Females
0–4	212	212
5–14	157	157
15–29	103	103
30–44	103	103
45–59	103	103
60–69	103	103
70–79	103	103
80+	103	103
Overall	134	133

The mortality of malaria was highest in children under five years, decreasing afterwards. Males and females were affected equally in all age groups. The overall mortality was the same in males and females.

The total number of deaths in 2002 was estimated to be about 44,000, far exceeding the numbers reported from the formal health system (2,125 deaths) in the same year [9]. This was expected since the formal health system registration did not capture all deaths in the population. Demographic calculations showed that all-cause death registration completeness in Sudan in 2002 was 4.8% [26]. The number of deaths in this study was higher than that estimated in the GBD 2002 [27], where country specific estimates were based on an analysis by Snow et al [28].

### DALYs

**Figure 3:**
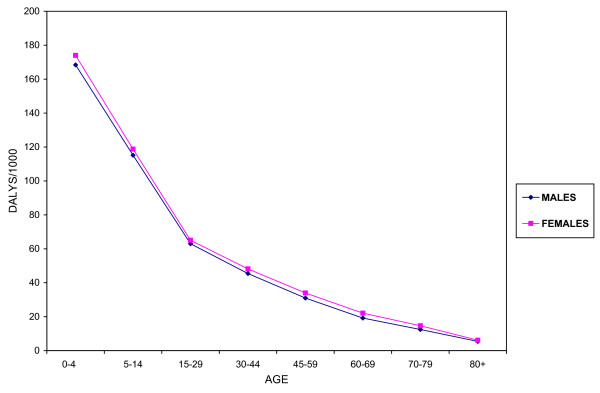
**DALYs lost per 1000 population due to malaria in Sudan in 2002 by age and sex**. In both males and females, the greatest loss of DALYs due to malaria was in children below 5 years. The burden dropped considerably in older ages. Females had a higher burden than males in all age groups.

Total DALYs and DALYs/1000 by age and sex are shown in Table 3 and Figure 3 respectively. Malaria accounted for a higher mortality burden than disability with 2,791,000 YLLS compared to 86,000 YLDs. This was due to the limited number of disabilities considered, as this study followed the mutually exclusive disease categorization of the GBD, where other consequences of malaria, such as renal complications, were included in other disease categories. An alternative approach to quantify the burden of malaria would consider, in addition to all direct complications, the burden of conditions for which malaria is a risk factor, such as low birth weight, that is attributed to malaria. In both males and females the highest burden of malaria was in children less than five years of age, decreasing considerably in older age groups. The fact that the youngest age group carried the highest burden resulted from the high mortality in this group as well as the loss of more years by deaths at younger age groups. This effect could be reduced by giving less weight to deaths in the young age group and by discounting years lost in the future, but the application of these processes remains controversial [23]. Despite that males had higher incidence and mortality rates, females lost more YLLs than males and consequently lost more DALYs. The higher loss in YLLs was due to the higher standard life expectancy of females.

**Table 3:** YLLs, YLDs and DALYs lost due to malaria in Sudan in 2002

	Males	Females	Total
YLLs (000)	1387	1404	2791
YLDs (000)	45	41	86
DALYs (000)	1432	1445	2877

The burden of the disabilities considered in this study (YLDs) was less than the burden of mortality (YLLs). Males had a higher disability burden than males while females had a higher mortality burden than males. Overall, females lost more DALYs than males.

The comparability of the results to results of similar studies in other countries was limited by the different methods, case definitions and disability weights used

### Role of computer modelling

DisMod II ensured that the estimates used in the final burden calculations were internally consistent. However, large discrepancies were previously found when an empirical data set was compared to DisMod II results and were attributed to data inaccuracies and past disease trends [29]. This emphasized the importance of judgement and expert opinion in processing the input and revising the output of the disease modelling software.

### Sources of uncertainty in the estimates

The validity of the results of this study depends on the validity of the data used in their synthesis. Although the best sources available were used, inadequate reports sometimes limited proper study appraisal. In some instances, no single study proved to be the best or most representative, and the results of all the available studies were summarized in one estimate.

The data sources used ranged from health facility based studies to nationally representative population based studies. The former were not representative of the population concerned and some population based studies, NGO surveillance data and national surveys did not cover all the country. Some figures were borrowed from studies in other African countries. However, non representative and non local studies were resorted to only where no local estimates could be found or were too unreliable to use.

Sudan states were classified by endemicity level although administrative boundaries between states did not coincide with boundaries between the different endemicity areas, according to a climate-based distribution model [30].

Most of the studies used were conducted before or after 2002. This could have affected the validity of the results because their use was based on the assumption of a stable epidemiological and demographic status, which cannot be assumed about malaria in Sudan.

Information about neurological sequelae due to malaria was found only for children less than nine years of age. The findings were generalized from these groups to all age groups due the lack of studies on the other age groups. Findings from northern Sudan were generalized to southern Sudan for the same reason.

Disability weights reflect societal preferences for health states. In spite of the extensive analysis in the GBD to ensure that the disability weights developed were highly reliable [1], they are expected to vary by the method of health state valuation, by the type of group conducting it and between different populations. Moreover, the case definitions used in the health state valuation of anaemia and neurological sequelae did not always match those of the studies used in the analysis. Uncertainties from these sources were carried over to the burden estimates. The use of extensive calculations in this study emphasized the need for quantifying uncertainty around the results. The complexity in the calculations of epidemiological estimates of malaria in Sudan arose from the complexity of malaria epidemiology. Sudan includes all levels of endemicity, and the variation in clinical presentation and age distribution with endemicity of malaria entailed extensive calculations to estimate age-sex specific rates.

### Data gaps

The search conducted in this study revealed the lack of representative and acceptably valid mortality data as well as representative data on case fatality, remission and complications of malaria in Sudan.

## Conclusion

The study revealed underreporting of malaria episodes and deaths to the formal health system, with the consequent underestimation of malaria burden. Children less than five years of age had the highest mortality rate and DALYs, emphasizing the known effect of malaria on this population group. Females lost more DALYs than males in all age groups, which altered the picture displayed by the incidence rates alone. The epidemiological estimates and DALYs calculations in this study form a basis for comparing interventions that affect mortality and morbidity differently, by comparing the amount of burden averted by them. The DALYs would mark the position of malaria among the rest of the diseases, if compared to DALYs due to other diseases. Uncertainty around the estimates should be considered when using them for decision making and further work should quantify this uncertainty to facilitate utilisation of the results. The data gaps revealed in this study call for more epidemiological research to fill them. This will allow regular updating of the burden estimates by replicating the calculations using updated input data.

## Competing interests

The author(s) declare that they have no competing interests.

## Authors' contributions

SIA designed the methodology, supervised and participated in the data collection and performed the calculations. EM and KMA revised and endorsed the methodology and the results. All authors read and approved the final manuscript.

## References

[B1] Murray CJL, Lopez AD (1996). The global burden of disease: a comprehensive assessment of mortality and disability from diseases, injuries and risk factors in 1990 and projected to 2020.

[B2] Global Burden of Disease in 2002: data sources, methods and results. http://www.who.int/healthinfo/paper54.pdf.

[B3] Malaria incidence estimate at country level for the year 2004 – Proposed estimates and draft report. http://www.who.int/malaria/docs/incidence_estimations2.pdf.

[B4] Sudan Roll Back Malaria consultative mission: essential actions to support the attainment of the Abuja targets. http://www.rbm.who.int/partnership/country/docs/EAfrica/reaping_sudan.pdf.

[B5] Sudan Ministry of Finance & National Economy, Central Bureau of Statistics: (1996). Population projections for Sudan 1993 – 2018 Khartoum.

[B6] WHO statistical information system. Life tables for WHO member states, Sudan – 2002. http://www.who.int/whosis/database/life_tables/life_tables_process.cfm?path=whos is,life_tables&language=English.

[B7] Sudan Federal Ministry of Health (2001). Annual Statistical Report 2000 Khartoum.

[B8] Sudan Federal Ministry of Health (2001). Multiple Indicators Cluster Survey 2000 Khartoum.

[B9] Sudan Federal Ministry of Health (2003). Annual Statistical Report 2002 Khartoum.

[B10] Malaria situation and the progress made in RBM implementation in southern Sudan. http://www.emro.who.int/rbm/presentations/irn04/South Sudan.ppt.

[B11] Ibrahim SM (2003). Decisive assessment of diagnostic staining methods of malaria in eight public and private laboratories, Khartoum area. Operational Research on Tropical Diseases: Final Report Summaries 1992 – 2000.

[B12] Malik EM, Eltahir HG, Ahmed ES (2005). Clinical and laboratory aspects of malaria among children with fever in a low transmission area of Sudan. East Mediterr Health J.

[B13] McCarthy MC, Haberberger RL, Salib AW, Soliman BA, El-Tigani A, Khalid IO, Watts DM (1996). Evaluation of arthropod-borne viruses and other infectious disease pathogens as the causes of febrile illnesses in Khartoum Province of Sudan. J Med Virol.

[B14] Siddig AA (2000). The use of the insecticide treated bed-nets as a method of malaria prevention and control in a Sudanese high seasonal malaria transmission area. MD thesis.

[B15] Creasy A, Hamad A, Elhassan IM, Giha H, Theander TG, Arnot DE (2004). 11 years of malaria surveillance and treatment in a Sudanese village highlight unexplained variation in disease susceptibility and outbreak severity. Parasitology.

[B16] Reyburn H, Mbatia R, Drakeley C, Bruce J, Carneiro I, Olomi R, Cox J, Nykya WMMM, Lemnge M, Greenwood BM, Riley EM (2005). Association of transmission intensity and age with clinical manifestation and case fatality of severe Plasmodium falciparum malaria. JAMA.

[B17] Giha AH, El-Ghazali G, Elqadir TM, A-Elbasit IE, Eltahir EM, Baraka OZ, Kheir MM, Adam I, Troye-Blomberg M, Theander TG, Elbashir MI (2005). Clinical pattern of severe P. falciparum malaria in Sudan in an area characterized by seasonal and unstable malaria transmission. Trans R Soc Trop Med Hyg.

[B18] Mathers CD, Vos T, Lopez AD, Salomon J, Ezzati M, editors (2001). National Burden of Disease Studies A Practical Guide.

[B19] Bondi FS (1992). The incidence and outcome of neurological abnormalities in childhood cerebral malaria: a long term follow up of 62 survivors. Trans R Soc Trop Med Hyg.

[B20] Zeidan Z, Kojal H, Habour M, Nowary K, Hashim F, Erikson B (2006). Clinical and epidemiological features of severe malaria in children in Sudan: hospital based prospective study. EMHJ.

[B21] Ataalla N (1994). Pattern of selected haematological changes in malaria infection in children in Khartoum state. MD thesis.

[B22] Elhassan AMA (1993). Erythropoietin level in anaemia due to Plasmodium falciparum malaria. MD thesis.

[B23] Schellenberg D, Kahigwa E, Sanz S, Aponte J, Mshinda H, Alonso P, Menendez C (2004). A randomized comparison of two anaemia treatment regimens in Tanzanian children. Am J Trop Med Hyg.

[B24] Agyepong IA, Kayonda JK (2004). Providing practical estimates of malaria burden for health planners in resource poor countries. Am J Trop Med Hyg.

[B25] Breman JG (2001). The ears of the hippopotamus: Manifestations, determinants and estimates of the malaria burden. Am J Trop Med Hyg.

[B26] Abdalla SIA (2005). The national burden of selected diseases and injuries in Sudan in 2002. MD thesis.

[B27] WHO health statistics and health information systems. Deaths and DALY estimates for 2002 by cause for WHO member states. http://www.who.int/healthinfo/statistics/bodgbddeathdalyestimates.xls.

[B28] Snow RW, Craig M, Deichmann U, Marsh K (1999). Estimating mortality, morbidity and disability due to malaria among Africa's non-pregnant population. Bull World Health Organ.

[B29] Kruijshaar ME, Barendregt JJ, Hoeymans N (2002). The use of models in the estimation of disease epidemiology. Bull World Health Organ.

[B30] MARA/ARMA project. Seasonality of malaria transmission in Sudan. http://www.mara.org.za/pdfmaps/SudSeasonality.PDF.

